# Rational Design and In Silico Evaluation of a Multiepitope Vaccine Targeting the uPAR for Cancer Immunotherapy

**DOI:** 10.1155/jimr/9126083

**Published:** 2025-10-16

**Authors:** Fahimeh Baghaei, Zahra Hemmat, Amir Taherkhani, Setareh Shojaei, Ali Teimoori

**Affiliations:** ^1^ Department of Oral and Maxillofacial Pathology, School of Dentistry, Hamadan University of Medical Sciences, Hamadan, Iran, umsha.ac.ir; ^2^ Research Center for Molecular Medicine, Institute of Cancer, Hamadan University of Medical Sciences, Hamadan, Iran, umsha.ac.ir; ^3^ Department of Virology, Faculty of Medicine, Hamadan University of Medical Sciences, Hamadan, Iran, umsha.ac.ir

**Keywords:** cancer, immunotherapy, multiepitope, TLR4, uPAR, vaccine

## Abstract

**Background:**

The urokinase plasminogen activator receptor (uPAR) plays a crucial role in cancer development and progression, making it an attractive target for immunotherapeutic strategies. This study aimed to develop a multiepitope vaccine targeting uPAR by incorporating T cell epitopes and a toll‐like receptor 4 (TLR4) agonist as an adjuvant.

**Methods:**

Immunoinformatics approaches were employed to predict and select immunogenic epitopes from the uPAR protein sequence. The selected epitopes were assembled into a multiepitope vaccine construct, including a TLR4 agonist derived from *Mycobacterium tuberculosis* as an adjuvant. The vaccine candidate underwent comprehensive in silico analyses, including antigenicity, allergenicity, physicochemical properties, and structural modeling. Molecular docking and molecular dynamics (MD) simulations were performed to evaluate the vaccine’s interaction with the TLR4 receptor and assess its structural stability. Also, vector design was performed using the SnapGene software, while immune response simulations were conducted with the C‐ImmSim server.

**Results:**

The multiepitope vaccine construct comprised five cytotoxic T lymphocyte (CTL) epitopes, five helper T lymphocyte (HTL) epitopes, and the TLR4 agonist adjuvant. The vaccine was predicted to be nonallergenic, antigenic, and soluble, with favorable physicochemical properties. Molecular docking analysis revealed a strong binding affinity between the vaccine and TLR4, with a docking score of −334.37kcal/mol. MD simulations demonstrated the structural stability and rigidity of the vaccine–TLR4 complex. The computational immune simulation predicted a strong vaccine response with lasting antibody production, robust cellular immunity, and immunological memory formation.

**Conclusion:**

The proposed multiepitope vaccine construct, consisting of carefully selected uPAR epitopes and a potent adjuvant, exhibits promising characteristics for inducing a robust immune response against cancer cells expressing uPAR. The favorable in silico results warrant further experimental validation and preclinical studies to assess the vaccine’s efficacy and potential as a cancer immunotherapeutic agent.

## 1. Introduction

The urokinase plasminogen activator receptor (uPAR), encoded by the PLAUR gene, occupies a central role in cancer development and progression, thus rendering it a significant focus for immunotherapeutic strategies [1–9]. Its influence extends across multiple dimensions of oncogenesis, encompassing cell proliferation, adhesion, invasion, metastasis, and the modulation of the tumor microenvironment. Robust evidence underscores the heightened expression of uPAR across diverse cancer types, correlating strongly with unfavorable prognostic indicators and aggressive tumor phenotypes [5–7]. uPAR’s distinctive expression pattern in malignant versus normal tissues is particularly noteworthy, rendering it a compelling target for therapeutic intervention. Immunotherapeutic modalities directed against uPAR, including chimeric antigen receptor (CAR) technology and antibody‐dependent cellular cytotoxicity (ADCC), exhibit notable efficacy in selectively targeting malignant cells while preserving healthy tissue integrity [3, 4, 8]. The advent of cross‐reactive antibodies targeting uPAR, capable of eliciting ADCC and serving as the foundation for antibody–drug conjugates (ADCs), underscores the translational promise inherent in this therapeutic target [8]. Additionally, the role of uPAR in sculpting the tumor microenvironment, particularly its impact on immune cell infiltration and function, is increasingly acknowledged as pivotal in driving cancer progression and therapy resistance. The observed correlation between uPAR expression and immune checkpoint activation in renal clear cell carcinoma (ccRCC) delineates a potential mechanism through which uPAR may foster an immunosuppressive milieu, thereby impinging upon the effectiveness of immunotherapeutic interventions [7]. This premise gains further traction from findings in melanoma, where heightened levels of uPAR + extracellular vesicles are linked to resistance against checkpoint inhibitor therapy [4]. Given this compelling evidence, the PLAUR gene, and by extension, uPAR, emerges as a pivotal immunotherapeutic target. Its distinctive expression profile in cancerous tissues vis‐à‐vis regular counterparts and its involvement in pivotal oncogenic processes underscore its potential for optimizing cancer treatment outcomes. The pursuit of uPAR‐targeted therapeutic strategies, encompassing CAR‐T cells, ADCs, and monoclonal antibodies engineered for ADCC, presents a promising avenue for augmenting the efficacy of immunotherapeutic regimens and ultimately facilitating patient outcomes.

T cells are critical players in antitumor immunity, executing cancer cell eradication through diverse mechanisms. First, cytotoxic T lymphocytes (CTLs), characterized by CD8+ T cells, directly recognize and eliminate cancer cells displaying tumor‐associated antigens via interaction with major histocompatibility complex (MHC) class I molecules. CTLs induce apoptosis in targeted cancer cells by releasing cytotoxic granules containing perforin and granzymes [10, 11]. Second, T helper (TH) cells, particularly Th1 cells, support antitumor immunity by secreting cytokines like interferon‐gamma (IFN‐γ) and interleukin‐2 (IL‐2), augmenting CTL and natural killer (NK) cell cytotoxicity, promoting antigen presentation, and fostering an inflammatory milieu hostile to tumor growth [12, 13]. Additionally, T cells, notably CTLs, infiltrate solid tumors, exerting cytotoxic effects within the tumor microenvironment. Increased densities of tumor‐infiltrating lymphocytes (TILs), particularly CTLs, often correlate with improved prognosis and enhanced survival across various cancer types [13]. Furthermore, T cells participate in immunosurveillance, continuously monitoring the body for abnormal or transformed cells, including cancer cells, and eliminating them before detectable tumor formation [10]. Memory T cells (CD8+ and CD4+) are generated upon encountering cancer cells, facilitating rapid and robust immune responses upon subsequent encounters with the same cancer‐associated antigens, providing long‐term protection [11]. These diverse functions of T cells underpin many cancer immunotherapies, such as cancer vaccines, adoptive cell transfer (ACT) therapies, and checkpoint inhibitors, which aim to bolster or revive the antitumor T cell response [14, 15].

The investigation into T cell vaccines targeting VEGFR2 in advanced pancreatic cancer underscores their potential as an immunotherapeutic approach. Phase 1 trials of VXM01 reveal its safety, tolerability, and capacity to evoke VEGFR2‐specific effector T cell responses without dose‐limiting toxicities [16, 17]. These trials demonstrate a reduction in tumor perfusion and upregulation of antiangiogenic biomarkers, indicative of therapeutic impact. Subsequent trials employing a prime–boost strategy sustain and amplify T cell responses, suggesting prolonged immunological surveillance against tumor angiogenesis. However, the transient nature of antiangiogenic effects underscores the need for sustained immunological pressure. Correlations between preexisting T cell levels and therapeutic outcomes highlight the significance of patient‐specific immune landscapes. T cell vaccines show promise in eliciting specific, potent, and sustained antitumor responses with minimal toxicity, necessitating further optimization in dosing regimens and patient selection criteria. These findings underscore T cell vaccines’ potential in cancer immunotherapy, necessitating continued research to harness their therapeutic benefits fully.

In recent decades, multiepitope vaccines have emerged as one of the most promising treatments for controlling cancer cells [18]. These novel therapeutic vaccines represent the next generation of immunotherapy, leveraging the immune system against cancer. The selection of antigens, epitopes, adjuvants, and linkers is critical in multiepitope vaccine design, as these factors can significantly impact clinical outcomes [19]. In the current era, bioinformatics plays a pivotal role in streamlining drug design processes, reducing costs, risks, and time by facilitating the identification of optimal antigens and potential epitopes. Immunoinformatics, a branch of bioinformatics, offers valuable tools for biologists to predict immunogenic epitopes of target antigens [19].

While multiepitope vaccines offer promising benefits, their weak immunogenicity remains a significant challenge for clinical applications [18]. To address this limitation and enhance protective immunity, additional components known as adjuvants can be incorporated to bolster immune responses [18].

One effective strategy involves leveraging toll‐like receptor (TLR) agonists as adjuvants from various microbial origins. Notably, the TLR4 agonist derived from *Mycobacterium tuberculosis* stands out as a potent adjuvant for multiepitope cancer vaccines [20]. This particular TLR4 agonist demonstrates robust immunomodulatory effects on tumors and holds promise as an effective adjuvant in cancer treatment [20]. The 50S ribosomal protein L7/L12 from *Mycobacterium tuberculosis* (locus tag RL7_MYCTU) is a highly effective adjuvant in multiepitope vaccine constructs, mainly owing to its potent immunostimulatory properties. Critically, its ability to activate innate immunity occurs through TLR4, a receptor that has been extensively studied and validated for its pivotal role as a cancer vaccine adjuvant. As a potent TLR4 agonist, the L7/L12 protein initiates both MyD88 and TRIF‐dependent signaling pathways. This robust activation is crucial for the maturation and activation of dendritic cells (DCs), which are indispensable for bridging innate and adaptive immune responses and are key targets for enhancing cancer immunotherapies. Upon binding to TLR4, L7/L12 drives DC maturation and stimulates the production of proinflammatory cytokines such as TNF‐α, IL‐1β, and IL‐6. This significantly enhances the antigen‐presenting capacity of DCs and fosters a potent cellular immune response. Subsequently, these activated DCs effectively prime naive T cells, promoting the polarization of both CD4+ TH cells and CD8+ CT cells, leading to increased IFN‐γ secretion and enhanced T cell‐mediated cytotoxicity responses critical for both infectious disease and antitumor immunity [21]. The incorporation of this critical *Mycobacterium tuberculosis* L7/L12 agonist at the N‐terminal of multiepitope vaccine constructs markedly boosts immunogenicity by improving antigen presentation and stimulating comprehensive humoral and cellular immune responses. Furthermore, it contributes to favorable physicochemical properties such as vaccine solubility and stability [22]. Research by Peng et al. [22], which included molecular docking and dynamics simulations, confirms the strong and stable binding between the L7/L12 adjuvant and TLR4, underscoring its efficacy in activating innate immunity and ensuring sustained immune stimulation.

**Figure 1:**
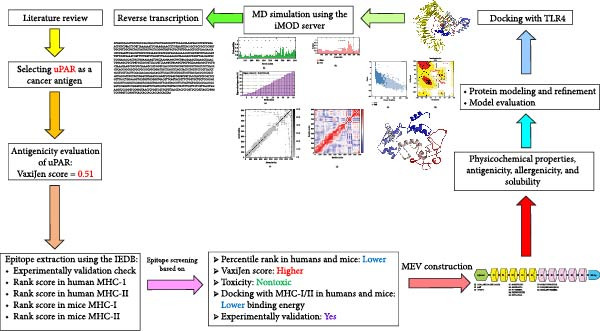
Graphical abstract of the study.

Our present study proposes a novel approach to developing a multiepitope vaccine against cancer. We utilized T cell epitopes derived from uPAR through immunoinformatics methodologies. Furthermore, we have augmented the vaccine construct by incorporating a TLR‐4 agonist as an adjuvant to enhance immune system stimulation [23]. A schematic overview of the entire study is presented in Figure 1.

## 2. Materials and Methods

### 2.1. The Meticulous Retrieval of the uPAR and Adjuvant Sequence

We strategically integrated the 50S ribosomal protein L7/L12 from *Mycobacterium tuberculosis* (locus tag RL7_MYCTU) in our vaccine construct design. This protein, recognized for its agonistic properties towards the TLR‐4/myeloid differentiation factor 2 (MD‐2) complex, was not just a random choice. Its incorporation was intended to leverage its inherent immunoadjuvant characteristics [24]. Multiple studies have underscored the strong binding affinity of this protein to TLR4, a pivotal pattern recognition receptor responsible for initiating innate immune responses upon detection of pathogen‐associated molecular patterns [25, 26].

### 2.2. Extraction of MHC‐I and MHC‐II Epitopes

The computational platform for predicting MHC class I and II epitopes was the Immune Epitope Database (IEDB) server, accessible at https://tools.iedb.org/mhci/ [27]. This platform, supported by the National Institute of Allergy and Infectious Diseases (NIAID), serves as an openly accessible repository aggregating experimental data on antibody and T cell epitopes studied across various species in the contexts of infectious diseases, allergies, autoimmunity, and transplantation. The IEDB also hosts tools for epitope prediction and analysis, demonstrating the use of advanced computational tools in our research. Its companion site, CEDAR (Cancer Epitope Database and Analysis Resource), funded by the National Cancer Institute (NCI), is dedicated explicitly to curating cancer epitopes.

The IEDB server computed the percentile rank for each epitope, considering the most frequent MHC class I and II alleles in human and mouse organisms (Table 1). Our study, while not evaluating the vaccine’s efficacy in mice, also assessed the binding affinity of the epitopes to MHC‐I and MHC‐II in mice. The objective was to design a vaccine containing epitopes demonstrating high binding affinity to MHC‐I and MHC‐II in human and murine models, a crucial factor in the vaccine’s potential effectiveness and our contribution to vaccine development.

**Table 1:** The most common MHC class I and class II alleles in humans and mice.

Organism	MHC‐I	MHC‐II
Human	HLA‐A^∗^02:01, HLA‐B^∗^07:02, HLA‐A^∗^24:02, HLA‐B^∗^44:02, HLA‐B^∗^08:01, HLA‐A^∗^01:01, HLA‐B^∗^35:01, HLA‐A^∗^11:01, HLA‐B^∗^15:02, HLA‐B^∗^40:01, HLA‐A^∗^03:01, HLA‐B^∗^18:01, HLA‐B^∗^51:01, HLA‐A^∗^26:01, HLA‐B^∗^58:01, HLA‐B^∗^27:05, HLA‐A^∗^68:01, HLA‐B^∗^38:01, HLA‐A^∗^31:01, and HLA‐B^∗^52:01	HLA‐DRB1 ^∗^01:01, HLA‐DRB1 ^∗^03:01, HLA‐DRB1 ^∗^04:01, HLA‐DRB1 ^∗^07:01, HLA‐DRB1 ^∗^08:01, HLA‐DRB1 ^∗^11:01, HLA‐DRB1 ^∗^13:01, HLA‐DRB1 ^∗^15:01, HLA‐DRB1 ^∗^16:02, HLA‐DPA1 ^∗^01:03‐DPB1 ^∗^02:01 (DP2), 2HLA‐DPA1 ^∗^02:01‐DPB1 ^∗^05:01 (DP5), HLA‐DPA1 ^∗^01:03‐DPB1 ^∗^04:02 (DP4), HLA‐DQA1 ^∗^01:01‐DQB1 ^∗^05:01 (DQ5), HLA‐DQA1 ^∗^03:01‐DQB1 ^∗^03:02 (DQ7), HLA‐DQA1 ^∗^05:01‐DQB1 ^∗^02:01 (DQ2), HLA‐DQA1 ^∗^06:02‐DQB1 ^∗^02:02 (DQ1), HLA‐DQA1 ^∗^01:02‐DQB1 ^∗^06:02 (DQ6), HLA‐DQA1 ^∗^03:03‐DQB1 ^∗^03:01, and HLA‐DQA1 ^∗^02:01‐DQB1 ^∗^02:02 (DQ2)

Mice	H‐2‐Dd, H‐2‐Kd, and H‐2‐Ld	H2‐IAD and H2‐IED

Abbreviation: MHC, major histocompatibility complex.

Furthermore, experimentally validated MHC‐I and MHC‐II epitopes of the uPAR were extracted from the IEDB database. These epitopes were deemed essential for the final vaccine formulation due to their potential to trigger immune responses. Subsequently, the development of the final multiepitope vaccine entailed integrating a combination of predicted and experimentally validated epitopes. Notably, among the experimentally validated epitopes, the most promising candidates underwent rigorous screening utilizing various algorithms, as outlined in the screening section.

### 2.3. Epitope Screening

Several complementary analyses were conducted to identify the most valuable epitopes for each MHC‐I and MHC‐II epitope previously extracted from the IEDB. The following protocol was implemented:

1. Initially, epitopes with a percentile rank exceeding one in humans were excluded from the study.
2. The remaining epitopes were evaluated for antigenicity using the VaxiJen v2.0 server (http://www.ddg-pharmfac.net/vaxijen/VaxiJen/VaxiJen.html) [28] and screened for potential toxicity using the ToxinPred server (http://crdd.osdd.net/raghava/toxinpred/) [29]. Epitopes predicted to be nonantigenic or toxic were eliminated from further analyses.
3. Epitopes that were experimentally validated or had a percentile rank less than 1.0 against murine alleles were retained for subsequent analysis.

At this stage, two distinct approaches were employed to select the most valuable epitopes.

a. The first method relied solely on prediction algorithms. These epitopes could be experimentally validated or not, but they were required to have a percentile rank score of less than 1 against murine alleles. Molecular docking analysis was performed between the epitopes and human MHC‐I alleles (HLA‐A0201 and HLA‐B3501) and murine MHC‐I alleles (H2‐Kd, H2‐Ld, and H2‐Dd) using the HPEPDOCK 2.0 server (http://huanglab.phys.hust.edu.cn/hpepdock/) [30]. Epitopes with binding energies less than or equal to −200 kcal/mol were retained for final screening based on the VaxiJen score.
b. The second approach focused on experimentally validated epitopes; the most valuable ones were selected based on the algorithms described in section (a). However, having a percentile rank score less than 1.0 against murine alleles was not critical for these epitopes.

### 2.4. Designing a Recombinant Vaccine Sequence

The process of epitope selection for MHC‐I and MHC‐II played a pivotal role in the development of the vaccine. A thorough evaluation methodology was implemented, encompassing various criteria, including percentile ranking in human and murine models, antigenicity scores obtained from the VaxiJen server, findings from molecular docking analyses, and confirmation through experimental validation. Epitopes demonstrating superior rankings across these parameters were identified as promising, given their potential to elicit targeted and robust immune responses. Consequently, these prioritized epitopes were earmarked as primary candidates for incorporation into a multiepitope vaccine formulation.

Following epitope selection, these segments were assembled using appropriate linker sequences. The choice of linkers was informed by those documented in databases specializing in multiepitope peptide vaccines and pertinent literature sources [23]. These linkers are critical in facilitating epitope presentation and ensuring proper spatial arrangement. Specifically, the adjuvant at the N‐terminus was linked to the initial cytotoxic T lymphocyte (CTL) epitope using the AEAAAKEAAAKEAAAKA linker sequence. The MHC‐I epitopes were interconnected using the AAY linker. The final CTL epitope was linked to the initial helper T lymphocyte (HTL) epitope via the GPGPG linker. This same linker was employed to connect the MHC‐II epitopes and to append the last HTL epitope to the *C*‐terminal His‐tag sequence.

A diverse set of 110 vaccine constructs exhibiting distinct primary sequences was randomly generated to evaluate the vaccine candidates comprehensively. Subsequently, this collection of vaccine designs was subjected to further rigorous modeling and assessment procedures.

### 2.5. Allergenicity and Antigenicity Prediction of Multiepitope Vaccines

The allergenicity assessment for the vaccine constructs was performed utilizing the AllerTOP server [31], which can be accessed at https://www.ddg-pharmfac.net/AllerTOP/. The AllerTOP algorithm employs auto‐cross covariance transformation to standardize protein sequences into uniform equal‐length vectors for accurate allergenicity prediction. Furthermore, the potential antigenicity of the primary sequences of the vaccine candidates underwent evaluation through the VaxiJen v2.0 server [28]. Notably, this server’s prediction accuracy ranges between 70% and 89%, contingent upon the organism under investigation.

### 2.6. Solubility and Physicochemical Characteristics of the Multiepitope Vaccine

The solubility assessment for the vaccine constructs was conducted using the SOLpro server available at http://scratch.proteomics.ics.uci.edu [32]. This server utilizes a two‐stage support vector machine (SVM) architecture to generate solubility predictions rapidly. Additionally, the ProtParam server accessible at http://web.expasy.org/protparam/ [33] was employed to calculate the recombinant vaccine sequences’ physical and chemical parameters. These parameters encompassed the instability index (where a value below 40 is indicative of protein stability), aliphatic index (with higher values suggesting enhanced thermal stability), theoretical isoelectric point (pI), half‐life in mammalian cells, and grand average hydropathy (GRAVY; where more positive values denote increased hydrophobicity of the amino acids).

### 2.7. Prediction of the Tertiary Structure and Model Refinement

The 3D structural prediction of the novel vaccine candidate was conducted utilizing the I‐TASSER server, accessible online at https://zhanggroup.org/I-TASSER/ [34]. I‐TASSER is a sophisticated computational framework for predicting protein structures and annotating structure‐based functionalities. This methodology employs a hierarchical approach, initially identifying structural templates from the Protein Data Bank (PDB) using a multiple threading technique called LOMETS. Subsequent stages involve assembling full‐length atomic models through iterative template‐based fragment assembly simulations. Insights into the functional attributes of the target protein are then gleaned by rethreading the generated 3D models through the BioLiP protein function database. Widely recognized as “Zhang server” or “UM–TBM,” I‐TASSER has consistently performed in protein structure prediction, securing top rankings in the community‐wide CASP (Critical Assessment of Structure Prediction) experiments from CASP7 to CASP15. Notably, the server was lauded as the top performer for function prediction in CASP9. Continuously evolving, the I‐TASSER server is actively developed to deliver the most precise protein structure and function predictions. This endeavor is driven by integrating cutting‐edge algorithms and methodologies within the field, ensuring ongoing advancements in accuracy and reliability.

To enhance the 3D structural model of the recombinant vaccine candidate, the Galaxy Refine server (https://galaxy.seoklab.org/cgi-bin/submit.cgi?type=REFINE) [35] was utilized. This online platform, supported by Galux Inc. and the Computational Biology Lab at Seoul National University’s Department of Chemistry, employs a robust refinement strategy validated during the CASP10 (Critical Assessment of Structure Prediction) experiment. The refinement begins with reconstructing side chain conformations and a thorough side chain repacking procedure. Subsequently, the overall structural model undergoes relaxation via molecular dynamics (MD) simulations. Notably, the CASP10 assessment recognized this method as the foremost approach for improving the quality of local structural features. Moreover, numerous studies attest to the consistent enhancement of global and regional structural quality when applying this refinement protocol to models generated by contemporary protein structure prediction servers [36].

### 2.8. Model Evaluation

To thoroughly assess the 3D structural model of the recombinant vaccine candidate, we employed two robust online resources: ProSA‐web (https://prosa.services.came.sbg.ac.at/prosa.php) [37] and PROCHECK (https://saves.mbi.ucla.edu/) [38, 39]. ProSA‐web is a comprehensive tool for evaluating protein structure quality and contextualizing scores within the broader landscape of known protein structures. Moreover, it identifies and highlights potential areas of concern within the 3D model via an interactive molecular viewer. This service meets validation needs encountered during protein structure determination processes, including *X*‐ray crystallography, NMR spectroscopy, and theoretical calculations.

In addition to ProSA‐web, the PROCHECK suite provides an intricate stereochemical analysis of the protein structure model. Its outputs comprise informative PostScript format plots and a thorough residue‐by‐residue report. These analyses enable an assessment of overall structural integrity by comparing against well‐refined structures at similar resolutions and identifying regions meriting further scrutiny. PROCHECK programs are indispensable for evaluating the quality of protein structures during the determination phase, preexisting structures and those derived from computational modeling approaches utilizing known templates.

### 2.9. Protein–Protein Docking

To investigate the potential molecular interactions between the recombinant vaccine candidate and the TLR4 receptor, molecular docking simulations were conducted utilizing the HDOCK server (http://hdock.phys.hust.edu.cn/) [40]. This web‐based platform offers a comprehensive array of tools for homology modeling, structure prediction, macromolecular docking, and biological data integration, facilitating robust and efficient protein–protein docking investigations. The HDOCK server employs a hybrid algorithm amalgamating template‐based and template‐free docking methodologies. The server autonomously predicts their potential interactions by providing amino acid sequences or PDB structures for the receptor and ligand molecules [41]. In this study, the crystal structure of TLR4 (PDB ID: 4G8A, resolution: 2.4 Å) served as the receptor structure for docking against the recombinant vaccine model. The template‐based docking facet of HDOCK utilizes structural insights from established protein–protein complexes to guide the docking procedure. At the same time, the template‐free component relies on energy‐based scoring functions and optimization algorithms to explore the conformational space and identify favorable binding modes. This hybrid approach enhances the precision and dependability of the docking forecasts, furnishing valuable insights into the prospective molecular interactions between the recombinant vaccine and its target receptor, TLR4. The PDBsum server, available at https://www.ebi.ac.uk/thornton-srv/databases/pdbsum/ [42], analyzed the molecular interactions between the vaccine and TLR4 residues in the docked complex.

### 2.10. MD Simulation

MD simulations were conducted using the iMOD server (iMODS, https://imods.iqf.csic.es/) [43] to explore the recombinant vaccine’s structural dynamics and flexibility. The iMODS, chosen for its fast and effective evaluation capabilities, employs advanced normal mode analysis (NMA) methodology. Its user‐friendly interface is compatible with web browsers and mobile devices allowing intuitive access to computational resources. The robust NMA implementation of iMODS enables the exploration of feasible trajectories among different conformational states, with interactive visualization and analysis for large macromolecular complexes [44]. This exhaustive examination provided valuable insights into the vaccine’s flexibility and potential functional implications. The efficient algorithms and user‐friendly interface of iMODS streamlined this pivotal investigation into the vaccine candidate’s structural biology.

### 2.11. Codon Optimization

Following the structural design of the vaccine, the reverse translation process was facilitated using the SMS server (https://www.bioinformatics.org/sms2/rev_trans.html) [45]. For codon optimization and quantitative analysis, the Java Codon Adaptation Tool (JCat) server (https://www.jcat.de/Start.jsp) [46] was employed. This tool enables the avoidance of cleavage sites for specific restriction enzymes and incorporates measures to prevent Rho‐independent transcription terminators in the codon‐optimized DNA sequence. The prediction algorithm for Rho‐independent transcription terminators utilized in this study was previously proposed by Ermolaeva et al. [47].

### 2.12. A Vector Design

For efficient expression in *Escherichia coli*, the target gene underwent codon optimization using the IDT Codon Optimization Tool. To enable directional cloning, the optimized gene sequence was engineered to incorporate specific NcoI and HindIII restriction sites. The pET28a expression vector (Novagen/Merck Millipore) served as the cloning and protein expression vehicle. This vector is advantageous due to its IPTG‐inducible T7 promoter and the presence of a C‐terminal 6 × His‐tag, which aids in subsequent protein purification.

### 2.13. In Silico Immune Simulation

The C‐ImmSim server, available at https://kraken.iac.rm.cnr.it/C-IMMSIM/ [48], was utilized to perform an immune simulation study aimed at analyzing immunogenicity and profiling the immune response. The simulation was carried out using the platform’s default settings.

## 3. Results

### 3.1. Prediction of T Cell Epitopes

The IEDB server was employed to predict potential epitopes of uPAR capable of binding to human and murine MHC‐I molecules with 9 and 10 residues, and MHC‐II molecules with lengths of 14 and 15 residues. Epitopes with a percentile rank exceeding 1, an antigenicity score below 0.5, or those predicted to pose potential toxicity were excluded from further consideration. The remaining epitopes were screened, validating any experimentally confirmed instances and predicting their binding energies to human and murine MHC‐I and MHC‐II alleles. Subsequently, the top‐ranked epitopes for MHC‐I and MHC‐II were identified based on the criteria outlined in the screening section.

Based on predictive parameters, four MHC‐I epitopes were deemed highly promising: GHPPLLPLL, VTYSRSRYL, FHNNDTFHFL, and MGHPPLLPL. Furthermore, the epitope HPPLLPLLL exhibited noteworthy attributes according to experimentally validated data (Table 2). Regarding MHC class II epitopes, DVQYRSGAAPQPGP demonstrated the highest predicted scores and was also experimentally validated. Additionally, SGRAVTYSRSRYLE and NSGRAVTYSRSRYL displayed significant predicted scores among the experimentally validated epitopes (Table 3).

**Table 2:** Candidate CD8+ epitopes.

MHC‐I alleles with percentile rank score < 1	Allele, with the most favorable percentile rank score	Percentile rank	Start	Epitope	VaxiJen score	Experimentally validated? (yes/no)	Docking scores with different alleles in human and mice (kcal/mol)					Binding to the MHC‐I in mice, based on percentile rank scores from IEDB? (score < 1, yes/no)
							HLA ‐ A0201	HLA ‐ B3501	H2‐Kd	H2‐Ld	H2‐Dd	
HLA‐A^∗^24:02, HLA‐B^∗^38:01	HLA‐B^∗^38:01	0.07	2	GHPPLLPLL	1.11	No	−214.73	−211.35	−239.03	−240.32	−202.77	Yes
HLA‐B^∗^52:01, HLA‐C^∗^08:01,	HLA‐B^∗^52:01	0.22	107	VTYSRSRYL	0.86	No	−263.80	−242.21	−239.38	−262.37	−240.97	Yes
HLA‐B^∗^38:01	HLA‐B^∗^38:01	0.04	181	FHNNDTFHFL	0.72	No	−275.60	−236.43	−256.16	−257.62	−229.95	Yes
HLA‐C^∗^08:01, HLA‐B^∗^52:01, HLA‐B^∗^51:01,	HLA‐C^∗^08:01	0.38	1	MGHPPLLPL	0.72	No	−213.61	−221.06	−235.71	−251.51	−212.82	Yes
HLA‐B^∗^51:01, HLA‐B^∗^38:01, HLA‐B^∗^07:02, HLA‐B^∗^35:01, HLA‐C^∗^08:01	HLA‐B^∗^51:01	0.06	3	HPPLLPLLL	1.09	Yes	−233.835	−234.123	−248.315	−244.983	−194.054	Yes

**Table 3:** Candidate CD4+ epitopes.

MHC‐II alleles with percentile rank score < 1	Allele with the most favorable percentile rank score	Percentile rank	Start	Epitope	VaxiJen score	Experimentally validated? (yes/no)	Docking scores with different alleles in humans and mice (kcal/mol)		Binding to the MHC‐I in mice, based on percentile rank scores from IEDB? (score < 1, yes/no)
							HLA‐DRB1_1101	H2‐IAd	
HLA‐DRB1 ^∗^07:01	HLA‐DRB1 ^∗^07:01	0.21	103	SGRAVTYSRSRYLE	1.11	YES	−296.57	−237.07	No
HLA‐DQA1 ^∗^03:03/DQB1 ^∗^03:01	HLA‐DQA1 ^∗^03:03/DQB1 ^∗^03:01	0.37	299	DVQYRSGAAPQPGP	0.55	YES	−237.60	−219.56	Yes
HLA‐DRB1 ^∗^07:01,	HLA‐DRB1 ^∗^07:01	0.8	102	NSGRAVTYSRSRYL	1.07	YES	−278.87	−254.54	No

### 3.2. Vaccine Construct

The multiepitope vaccine was formulated based on the highly prioritized epitopes identified for the uPAR protein. Five MHC‐I epitopes extracted from Table 2 and three distinct MHC‐II epitopes from Table 3 were chosen for integration into the final construct. The sequences SGRAVTYSRSRYLE and DVQYRSGAAPQPGP were duplicated within the vaccine, resulting in five CTL epitope regions and five HTL epitope regions.

**Figure 2:**
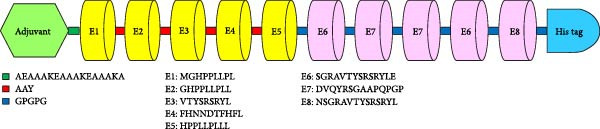
The structural layout of the final vaccine construct.

An adjuvant sequence (MAKLSTDELLDAFKEMTLLELSDFVKKFEETFEVTAAAPVAVAAAGAAPAGAAVEAAEEQSEFDVILEAAGDKKIGVIKVVREIVSGLGLKEAKDLVDGAPKPLLEKVAKEAADEAKAKLEAAGATVTVK), was positioned at the N‐terminus of the construct. Additionally, a hexahistidine tag was appended to the C‐terminus. The adjuvant was linked to the initial MHC‐I epitope via the AEAAAKEAAAKEAAAKA linker, known for its ability to form an alpha‐helical structure, thereby segregating the adjuvant as an independent domain. The MHC‐I and MHC‐II epitopes were interconnected using the AAY and GPGPG linker sequences, respectively. A total of 110 vaccine constructs, each comprising 311 amino acid residues were assembled, as depicted in Figure 2.

### 3.3. Allergenicity, Antigenicity, Physicochemical Characteristics, and Solubility of the Best Vaccine Construct

From an initial pool of 110 primary vaccine candidate sequences, an extensive in silico screening process was performed. Each candidate was meticulously evaluated based on stringent criteria, including its predicted allergenicity, antigenicity, solubility, and overall physicochemical attributes. This rigorous analysis aimed to identify the most promising vaccine sequence that exhibited favorable immunological and biophysical properties. The most favorable vaccine sequence was demonstrated to possess a highly desirable profile, being confirmed as nonallergenic. Its stability and suitability for development were further supported by an instability index of 30.74, an aliphatic index of 78.65, a theoretical pI of 6.84, a predicted half‐life of 30 h in mammalian cells, a GRAVY score of −0.237, a VaxiJen score of 0.61, and a high solubility probability of 0.97. These collective attributes demonstrate that the designed vaccine construct sufficiently meets the necessary criteria, thus warranting further experimental validation and preclinical studies.

### 3.4. Homology Modeling, Model Refinement, and 3D Structure Evaluation

The I‐TASSER server was utilized for protein modeling, and the most reliable model was selected based on a C‐score of −3.97. The C‐score serves as a confidence metric, assessing the quality of predicted models by I‐TASSER. It is derived from the significance of threading template alignments and the convergence parameters of structure assembly simulations. Typically falling within the range of [−5, 2], a higher C‐score indicates a model with greater confidence, and conversely, a lower score suggests lower confidence. Subsequently, the 3D model underwent refinement using the Galaxy Refine server, followed by validation steps.

**Figure 3:**
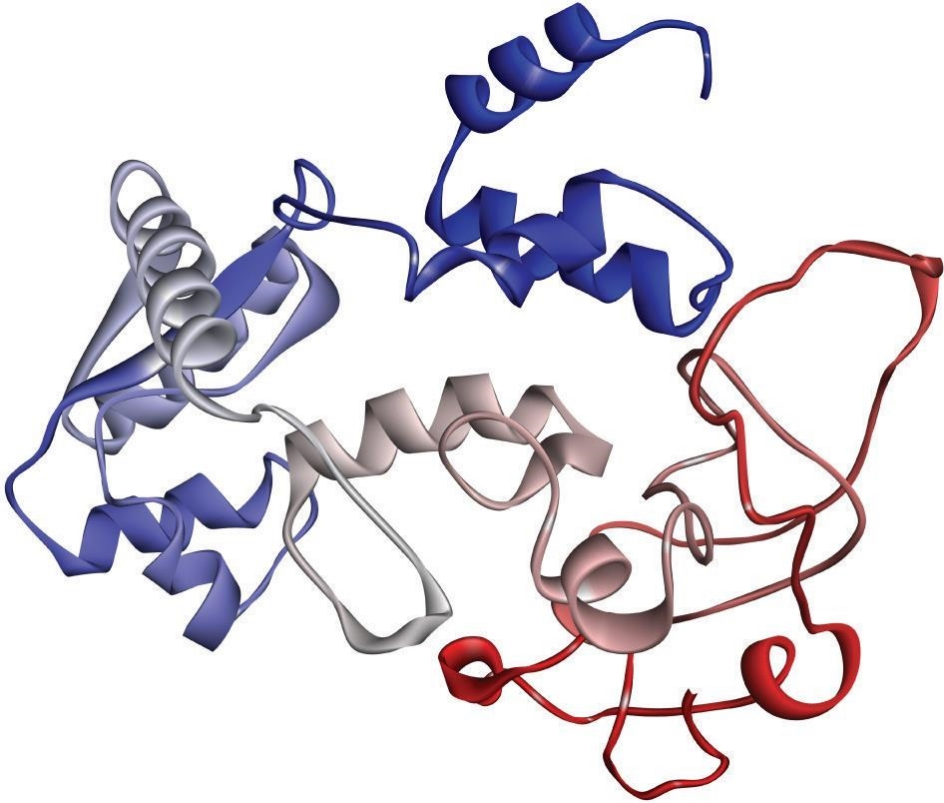
The three‐dimensional structure of the recombinant multiepitope vaccine showcases a color gradient along the chain, transitioning from blue at the N‐terminus, where the adjuvant is situated, to red at the C‐terminus.

For the structural evaluation of the refined model (Figure 3), the ProSA‐web and PROCHECK online tools were employed. The ProSA‐web analysis indicated similarity between the 3D structure of the vaccine and native proteins whose structures have been elucidated using *X*‐ray or NMR techniques, with a computed *Z*‐score of −6.7 for the refined model (Figure 4a). Furthermore, analysis of the Ramachandran plot obtained from the PROCHECK server revealed that 67.9% of residues resided in the most favorable regions, 23% in additional allowed regions, 5.3% in generously allowed regions, and 3.7% in the disallowed areas (Figure 4b). Figure S1 illustrates the overlay of the multiepitope vaccine structure prior to and following model refinement.

Figure 4(a) The three‐dimensional model of the vaccine construct was evaluated using ProSA‐web after refinement, resulting in a *Z*‐score of −6.7. (b) After refinement, the evaluation of the three‐dimensional model of the vaccine construct using the Ramachandran plot indicates the distribution of residues: 67.9% in favored regions, 23% in additional allowed regions, 5.3% in generously allowed regions, and 3.7% in disallowed regions.

**Figure:**
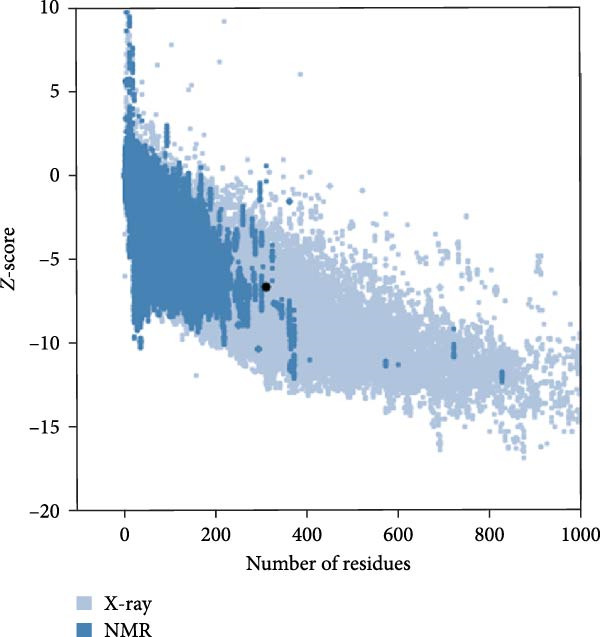
(a)

**Figure:**
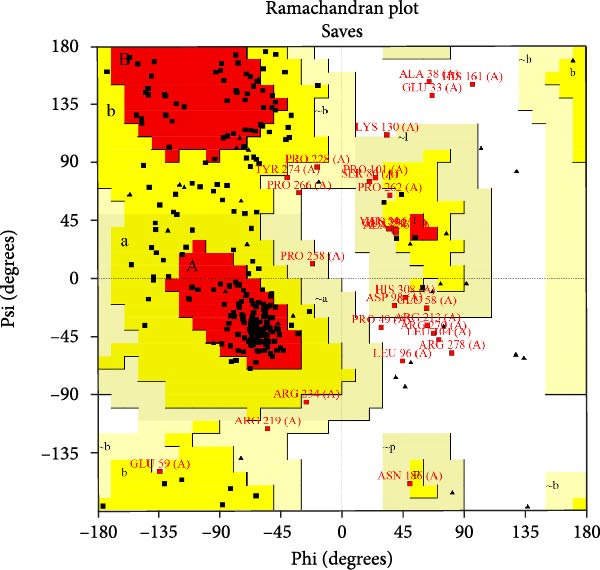
(b)

### 3.5. Molecular Docking

**Figure 5:**
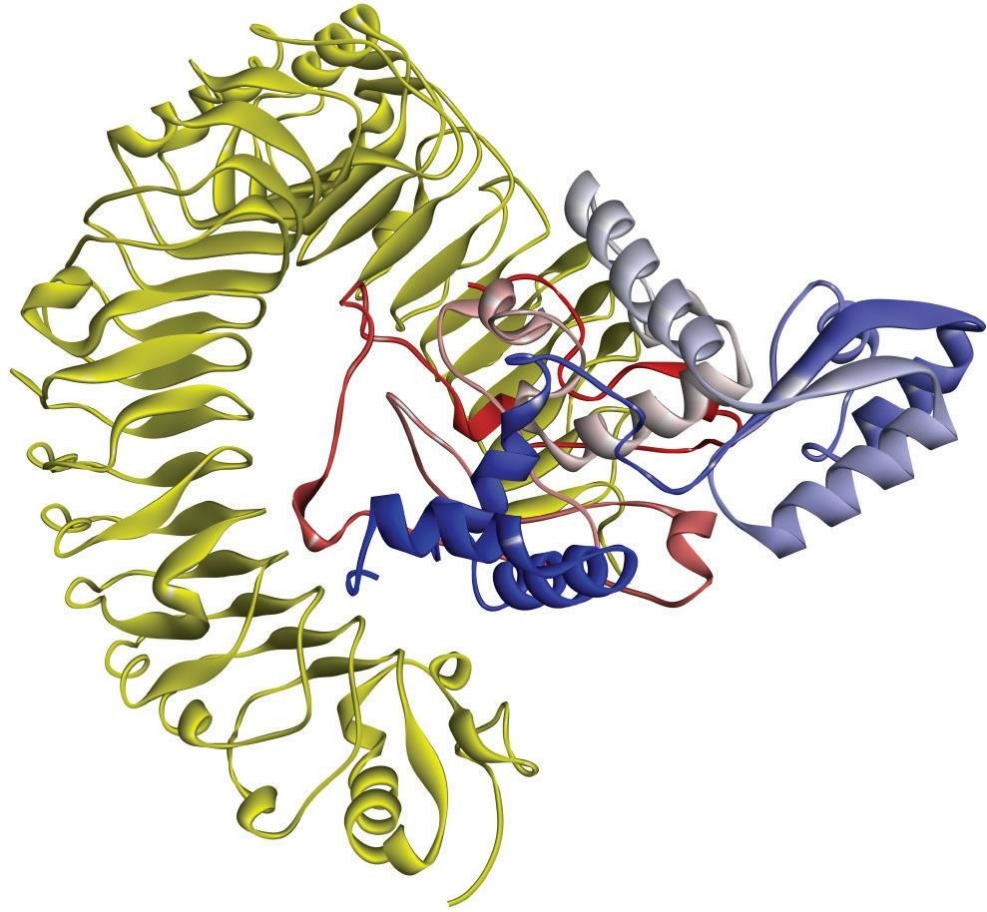
The docked complex illustrates the interaction between the vaccine construct (color gradient from blue at the N‐terminus to red at the C‐terminus) and TLR4 (depicted in yellow).

**Figure 6:**
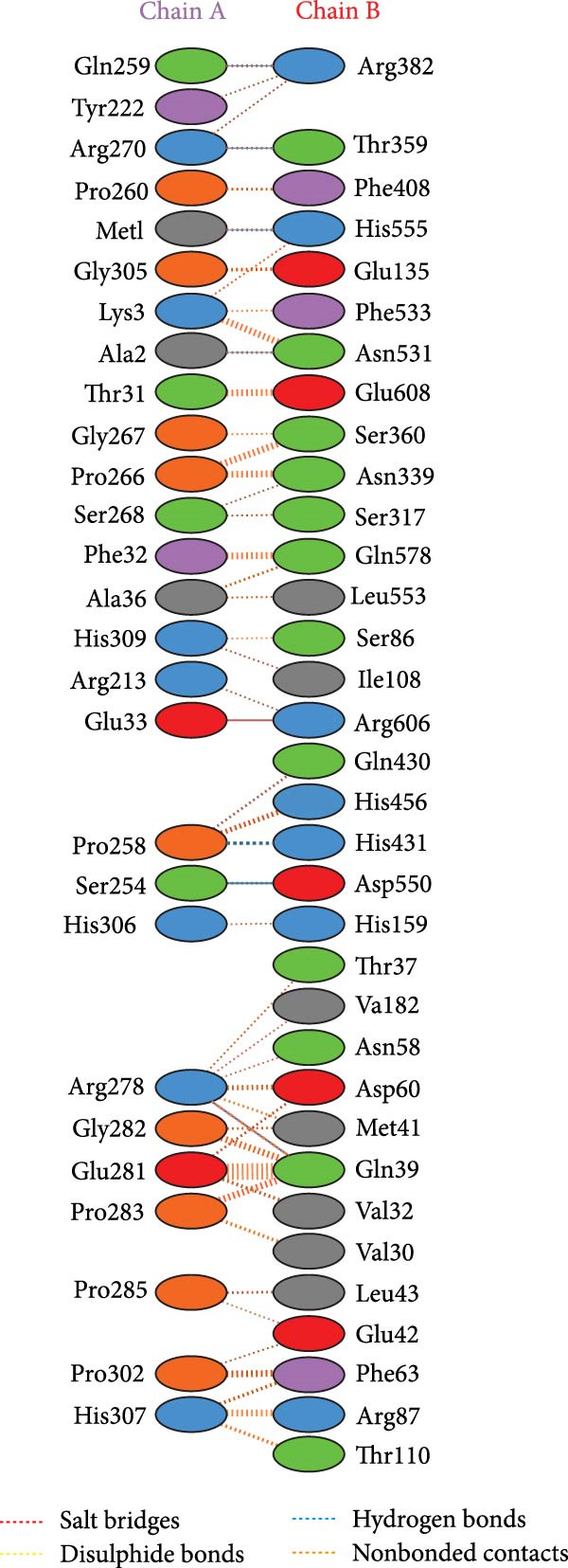
Interactions between the multiepitope vaccine and TLR4, visualized after molecular docking analysis using PDBsum. Hydrogen bonds and salt bridges are shown as blue and red dashed lines, respectively. TLR4, toll‐like receptor 4.

The HDOCK server evaluated the binding affinity between the recombinant vaccine candidate and TLR4. This computational docking platform generated 100 models depicting potential binding poses between the receptor (TLR4) and the ligand (multiepitope vaccine construct). Each of these poses underwent evaluation and scoring using an energy‐based scoring function. The top‐ranking docking model was selected based on its lowest binding energy value (docking score of −334.37kcal/mol) and a high confidence score of 98%. This optimal docking pose, delineating the predicted binding mode of the vaccine with TLR4, was further examined using the molecular visualization software Discovery Studio Visualizer (Figure 5). Figure 6 illustrates interactions between the multiepitope vaccine and TLR4. Details on hydrogen and salt bridges, including distances, are provided in Table 4.

**Table 4:** Hydrogen and salt bridges bonding residues between the multiepitope vaccine and TLR4 in the docked complex.

MEV	TLR4	Bond length (Å)
(A) Hydrogen‐bonding residues		
Gln259	Arg382	2.64
Arg270	Thr359	2.87
Met1	His555	3.07
Ala2	Asn531	3.34
Pro258	His431	3.14
Ser254	Asp550	3.06
Arg278	Gln39	2.02
(B) Salt bridges residues		
Glu33	Arg606	3.96

Abbreviation: TLR4, toll‐like receptor 4.

### 3.6. MD Simulation

MD simulations were conducted using the iMODs server to assess the stability and conformational dynamics of the docked recombinant vaccine–TLR4 complex. Deformability analysis (Figure 7a) indicated minimal distortion within the complex structure, supported by observing a low number of hinges. This finding was further substantiated by the *B*‐factor plot (Figure 7b), where low values corresponding to root mean square (RMS) fluctuations suggested high stability of the docked recombinant vaccine–TLR4 complex. Additionally, a relatively high eigenvalue of 2.430060 × 10^−5^ (Figure 7c) implied that substantial energy would be required to deform the vaccine–TLR4 complex, underscoring its structural rigidity. The covariance matrix (Figure 7d) delineates correlated motions between residue pairs, with red, white, and blue denoting positively correlated, uncorrelated, and anticorrelated motions, respectively. Furthermore, visualization of the elastic network model (Figure 7e) aids in identifying regions of varying flexibility within the complex, with darker gray indicating higher protein stiffness in specific areas.

Figure 7Utilizing the iMODS server, molecular dynamics simulations were performed, yielding the following analyses: (a) deformability plot, (b) *B* factor plot, (c) eigenvalue assessment of the recombinant vaccine–TLR4 complex, (d) covariance matrix illustrating correlations between pairs of residues, with red indicating correlated motion, white representing uncorrelated motion, and blue depicting anticorrelated motion, and (e) elastic network model, where darker gray signifies more rigid springs.

**Figure:**
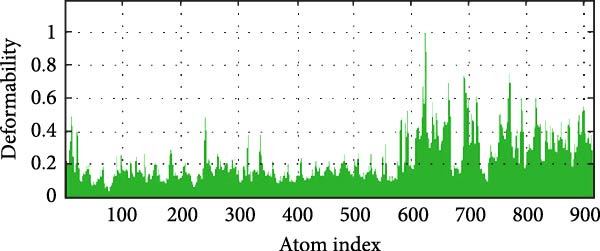
(a)

**Figure:**
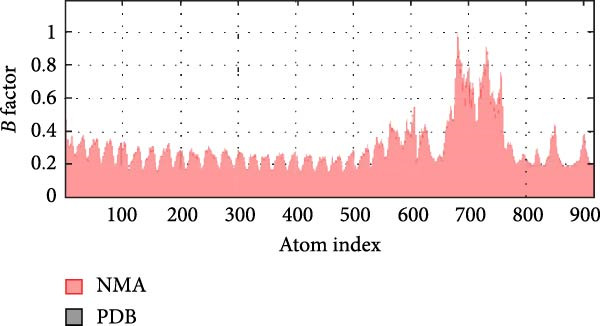
(b)

**Figure:**
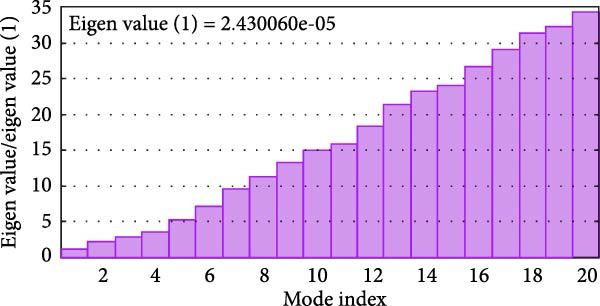
(c)

**Figure:**
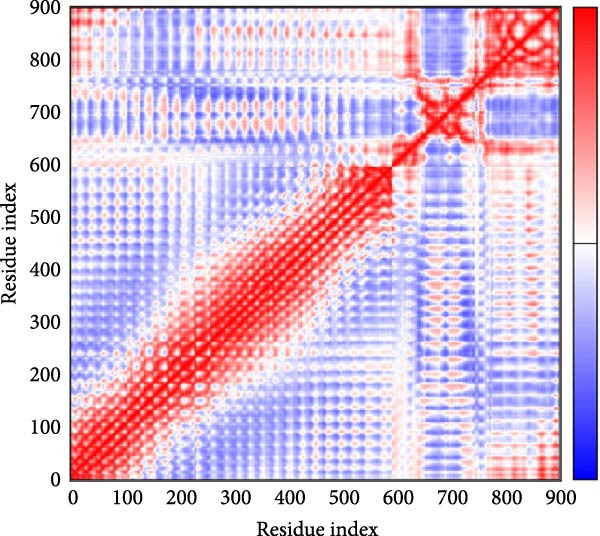
(d)

**Figure:**
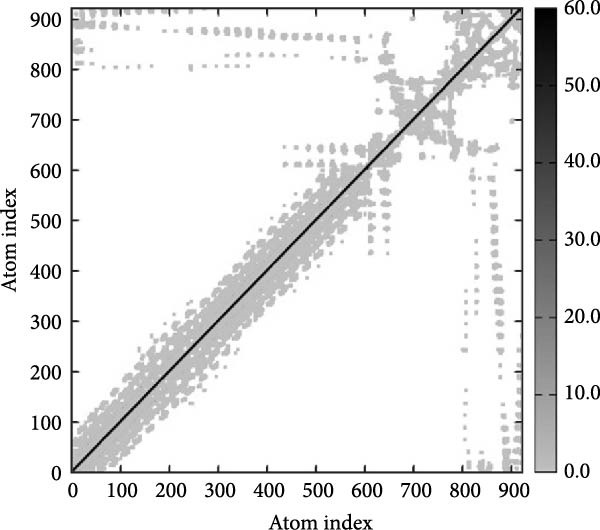
(e)

### 3.7. In Silico Reverse Transcription, Codon Optimization, and Vector Design

**Figure 9:**
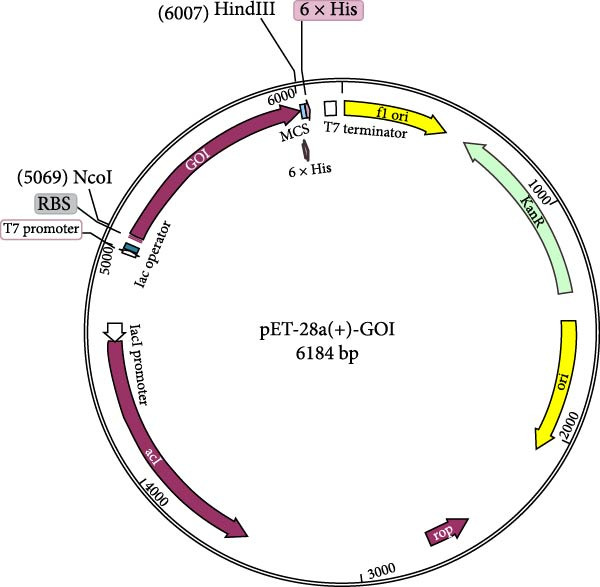
Plasmid map of the pET‐28a(+)‐GOI recombinant expression vector. The map illustrates the construct designed for high‐level expression of the multiepitope vaccine (gene of interest [GOI], shown in purple) in *E. coli* host systems. The GOI is cloned into the pET‐28a(+) backbone, with expression driven by the T7 promoter and controlled by a lac operator for inducibility. The vector also contains a 6 × His tag for affinity purification of the expressed protein and a kanamycin resistance gene (KanR) for bacterial selection. The positions of the HindIII (6007 bp) and NcoI (5069 bp) restriction enzyme sites are indicated.

The amino acid sequence of the rationally designed novel multiepitope vaccine underwent reverse translation into a DNA sequence using the SMS web server. Subsequently, to enhance protein expression levels in the *E. coli* K12 strain, the nucleotide sequence underwent codon optimization utilizing the JCat web server. Following optimization, an optimal Codon Adaptation Index (CAI) value of 1.0 was achieved, accompanied by a GC content of 56.20%. A CAI value approaching 1.0 indicates higher expression efficiency in the desired host organism [46]. With these optimized sequence parameters, a high recombinant protein expression level for the designed multiepitope vaccine construct is anticipated in the *E. coli* K12 expression strain. Figure 8 displays the multiepitope vaccine’s linear amino acid and optimized DNA sequences. Integration of the optimized sequence into the pET28a(+) vector was successfully performed and visualized using the SnapGene program (Figure 9).

Figure 8Recombinant multiepitope vaccine: linear formula depicting (a) amino acid and (b) optimized DNA sequences.

**Figure:**
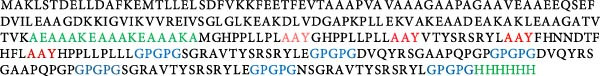
(a)

**Figure:**
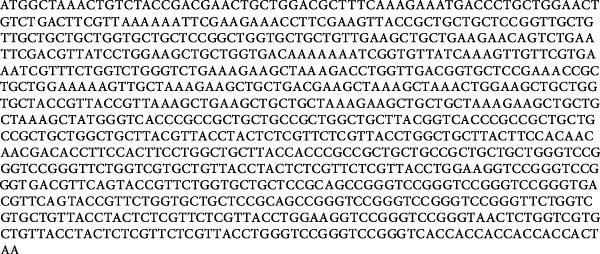
(b)

### 3.8. Vaccine Immune Simulation

The in silico vaccine simulations demonstrate a robust immune response following antigen administration. As depicted in Figure 10a, the antigen rapidly decreases, coinciding with a significant rise in antibody titers, particularly IgM and IgG, indicating successful humoral immunity. Figure 10b further elaborates on the B cell population dynamics, showing an initial expansion of total B cells, followed by a differentiation into memory B cells and various IgG isotypes, confirming a sustained adaptive response. Concurrently, TH cells (Figure 10c) exhibit a strong activation and proliferation phase, with a notable increase in active and duplicating TH cells, essential for coordinating both humoral and cellular immunity. CT cells (Figure 10d) also show activation, suggesting the induction of cell‐mediated immunity capable of targeting cancer cells. DC populations (Figure 10e) showed initial activation followed by steady‐state levels around 200 cells/mm^3^. Finally, the cytokine profile (Figure 10f) revealed the transient upregulation of key proinflammatory and regulatory cytokines such as IFN‐γ and IL‐2, which are indicative of a well‐orchestrated immune activation and subsequent resolution.

Figure 10Immune response dynamics following immunization. Time‐course analysis showing (a) antibody titers, (b) B cell populations, (c) T helper cell subsets, (d) cytotoxic T cell states, (e) dendritic cell populations, and (f) cytokine production over 35 days postimmunization.

**Figure:**
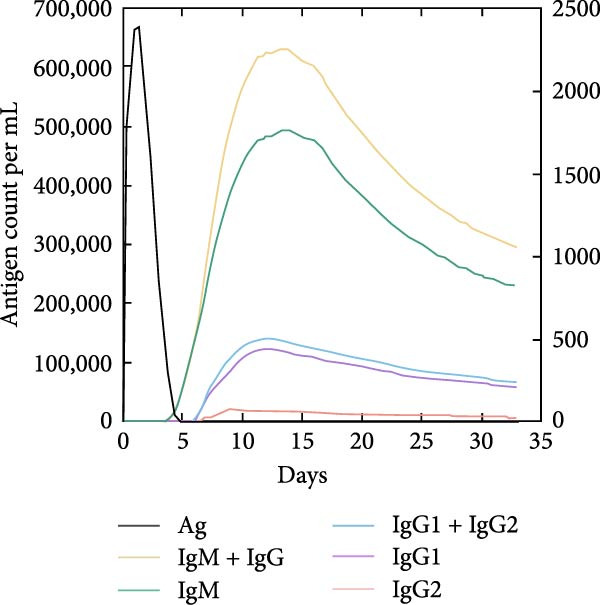
(a)

**Figure:**
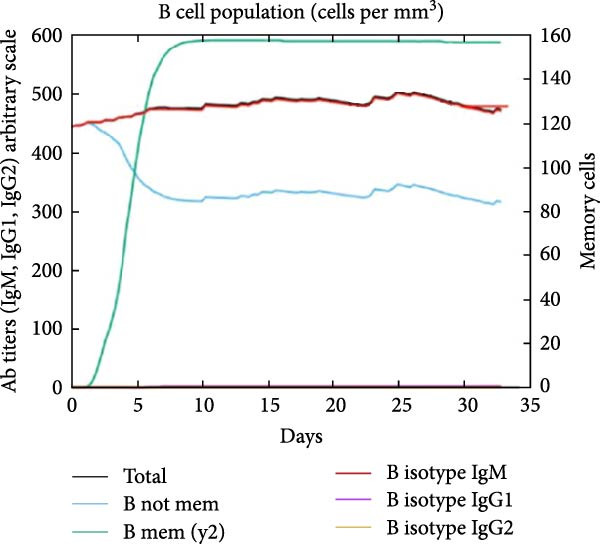
(b)

**Figure:**
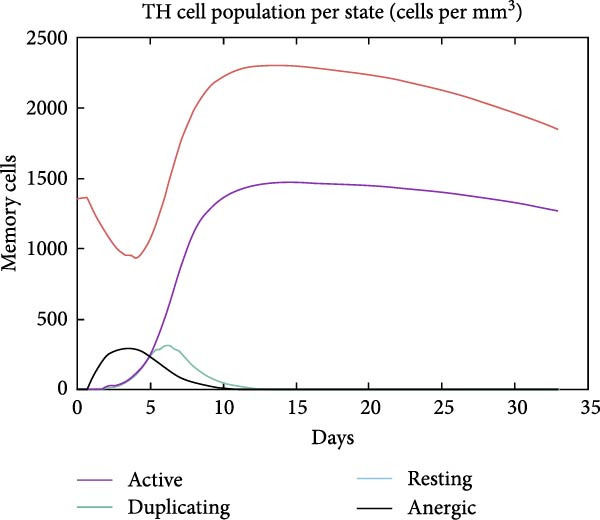
(c)

**Figure:**
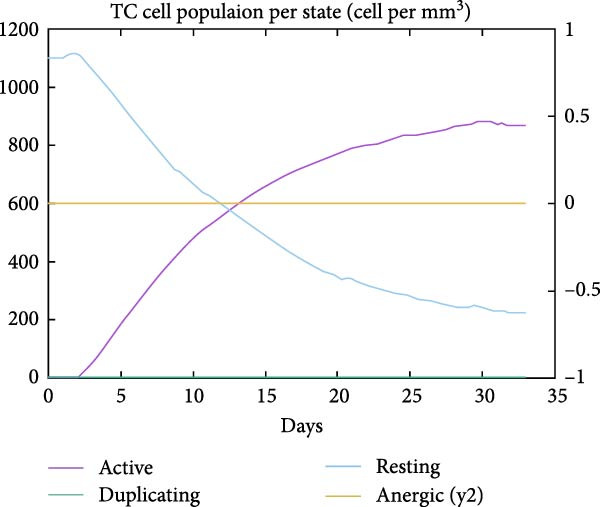
(d)

**Figure:**
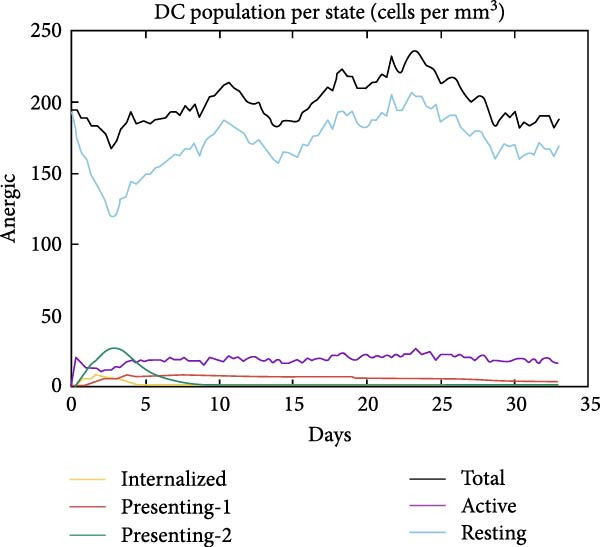
(e)

**Figure:**
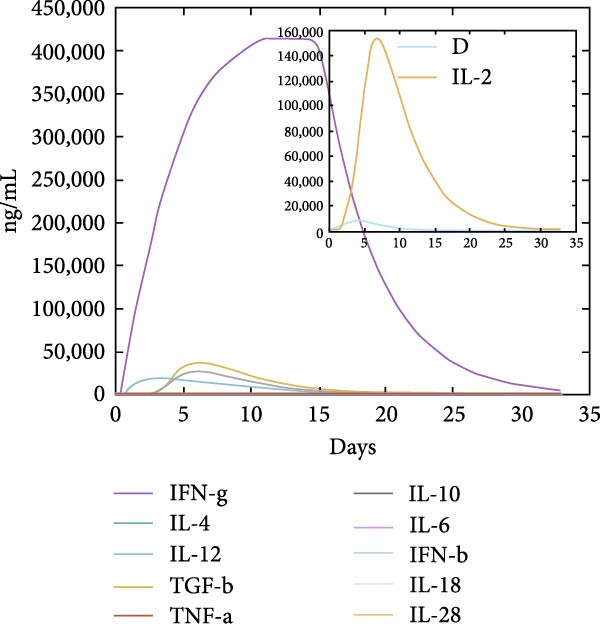
(f)

## 4. Discussion

The uPAR is widely recognized as a pivotal factor in cancer pathogenesis, influencing critical processes, including tumor invasion, metastasis, angiogenesis, cell proliferation, and apoptosis [6, 49–52]. Through its binding with urokinase‐type plasminogen activator (uPA), uPAR instigates the conversion of plasminogen to plasmin, initiating a cascade resulting in the degradation of the extracellular matrix (ECM), thereby facilitating tumor invasion and metastasis [49, 50]. In addition to its enzymatic functions, uPAR interacts with various cell surface receptors, such as integrins, modulating cell adhesion, migration, and signaling pathways conducive to tumor advancement and resistance to apoptosis [6, 49, 52]. Extensive documentation underscores the prognostic significance of uPAR expression in cancer, with elevated levels correlating with adverse survival outcomes, heightened tumor aggressiveness, and increased metastatic potential [49, 51, 52]. This correlation underscores uPAR’s potential as a therapeutic target. Diverse research endeavors have explored multiple modalities for targeting uPAR, encompassing the development of small‐molecule inhibitors, monoclonal antibodies, and other agents designed to impede the interaction between uPAR and uPA or disrupt the receptor‐initiated signaling cascades [6, 49, 50]. Notably, monoclonal antibodies directed at uPAR offer a promising therapeutic avenue, demonstrating efficacy in preclinical cancer models by inhibiting tumor growth and metastasis [49]. Furthermore, the disruption of uPAR’s interaction with integrins and ECM proteins, thereby hindering downstream signaling pathways implicated in tumor progression, represents another feasible therapeutic approach [50, 51]. Innovative strategies, such as nanoplatforms carrying therapeutic payloads and photodynamic and photothermal therapy platforms targeting uPAR, have also emerged providing novel means of delivering treatments directly to tumor cells while minimizing collateral damage to healthy tissues [6].

The immune system is crucial in managing malignancy because it detects and responds to cancer cells expressing tumor‐associated antigens presented through MHC molecules [53]. Multiepitope vaccines are engineered to elicit or augment a robust T cell response capable of recognizing and eliminating cancer cells. Consequently, the judicious selection of antigenic targets is critical in rationalizing effective cancer vaccine candidates [23]. In this current study, we devised a recombinant vaccine to stimulate the immune system to target uPAR directly. Recognizing the pivotal role of T cells in targeting malignant cells, we employed a comprehensive immunoinformatic approach to identify the most immunogenic epitopes derived from the uPAR. The selected epitopes demonstrated high binding affinities to both human MHC class I and II molecules. Notably, a subset of these epitopes also exhibited robust interactions with murine MHC class I and II, facilitating potential preclinical evaluation in relevant animal models.

In addition to meticulous antigenic target selection, the choice of adjuvant plays a pivotal role in determining the efficacy of recombinant vaccine candidates. Various adjuvants, such as Montanide formulations, aluminum salts, and TLR agonists, have been scrutinized by researchers in diverse investigations [23, 24, 54–58]. In this current study, we opted for a TLR4 agonist, recognized for its potent immunostimulatory properties, to elicit a robust immune response. TLR4 stands out among TLR family members due to its ability to simultaneously activate cellular and humoral immune responses [23].

The present vaccine construct is characterized by two functionally separate modules that are joined by an AEAAAKEAAAKEAAAKA linker. At the N‐terminus of this linker, a specific TLR4 agonist has been positioned. The role of this agonist is to activate TLR4, leading to DC maturation, proinflammatory cytokine production, and robust T cell‐mediated immune responses. This enhances the overall immunogenicity, protective efficacy, and stability of the vaccine [21, 22]. Conversely, at the C‐terminus of the linker, the main immunogenic component of the vaccine is situated. This part is comprised of carefully selected uPAR epitopes. The purpose of these epitopes is to be presented by MHC‐I and MHC‐II molecules on antigen‐presenting cells. This presentation is designed to specifically prime and activate cytotoxic CD8+ T cells (with guidance from CD4+ TH cells). These activated T cells will then recognize and specifically target cancer cells that overexpress uPAR on their surface. Therefore, it is crucial to emphasize that uPAR itself is not stimulated by the vaccine; instead, it serves as the direct target for immune system attack, leading to the specific eradication of uPAR‐positive cancer cells. This strategic separation and specific targeting mechanism is designed to mitigate the risks associated with generalized inflammation or the inadvertent promotion of tumor progression, focusing instead on a potent and selective anticancer effect.

The novelty of this approach lies in the comprehensive and stringent in silico pipeline that was employed for epitope selection. This pipeline goes beyond standard prediction methods by integrating multiple prediction algorithms, rigorous antigenicity and allergenicity assessments, and detailed structural analyses (e.g., molecular docking and dynamics simulations with TLR4). This multilayered screening ensures that highly immunogenic and safe epitopes are selected. Furthermore, the vaccine construct has been augmented by incorporating a TLR4 agonist as an adjuvant to enhance immune system stimulation. The relevance of the chosen methods is underscored by their ability to significantly reduce the time and cost associated with traditional vaccine development, offering a rational and efficient design strategy, particularly for complex targets like uPAR, and enabling the rapid identification of up‐and‐coming vaccine candidates for subsequent experimental validation.

Incorporating appropriate linker sequences is crucial for the optimal performance of the recombinant protein vaccine candidate. Consequently, our multiepitope subunit vaccine was constructed by strategically combining the adjuvant, CTL, and HTL epitopes with rationally designed linker moieties. Specifically, we utilized the following linkers: (EAAAK)×3, AAY, and GPGPG. The rigid (EAAAK)×3 linker was employed to confer structural stability and maintain a fixed spatial separation between the adjuvant and epitope components [59, 60]. The AAY linker was included to enhance epitope processing and presentation by increasing the accessibility of the C‐terminus of CTL epitopes for molecular interactions involved in antigen presentation pathways. Similarly, the GPGPG linkers were strategically incorporated to connect the HTL epitopes, promoting an optimal HTL response [61, 62].

The physicochemical and structural attributes of the designed recombinant vaccine construct were comprehensively evaluated using multiple immunoinformatic tools. The results revealed an instability index of 30.74, classifying the protein as stable. Furthermore, the primary amino acid sequence was predicted to be nonallergenic. Additional computed parameters included an aliphatic index of 78.65, a theoretical isoelectric point of 6.84, a projected half‐life of 30 h in mammalian cells, a GRAVY score of −0.237, a VaxiJen antigenicity score of 0.61, and a solubility probability of 0.97. The favorable instability index suggests that the multiepitope vaccine construct will likely exhibit stability across various temperatures. Moreover, the negative GRAVY score indicates a hydrophilic nature, facilitating favorable interactions with the surrounding aqueous environment. With a high solubility probability of 0.97, the recombinant vaccine protein is anticipated to demonstrate sufficient solubility upon overexpression in an *Escherichia coli* host system. Notably, the vaccine’s predicted antigenicity and nonallergenic profile bode well for its ability to elicit a robust immune response while mitigating the risk of adverse allergic reactions.

The MD simulation analysis demonstrated that the recombinant vaccine–TLR4 complex exhibited minimal conformational distortion over the simulation trajectory. Furthermore, the relatively high eigenvalue of 2.430060 × 10^−5^ obtained from the calculations suggests that substantial energy would be required to deform the vaccine–TLR4 complex, underscoring its structural rigidity and stability. These findings provide compelling evidence for the structural robustness of the designed vaccine construct in complex with its adjuvant component, a critical factor for maintaining its intended immunogenic properties.

To facilitate high‐level expression of the novel vaccine candidate in the *E. coli* host system, codon optimization was performed on the coding sequence [63]. The analysis revealed that the designed construct exhibited a solubility score of 100%, indicating a remarkable propensity for overexpression in a soluble form upon production in the *E. coli* expression host. This favorable solubility profile is a critical determinant for the successful overexpression and purification of the recombinant multiepitope vaccine, enabling its subsequent evaluation and characterization.

## 5. Conclusion

The present study described the rational design and comprehensive in silico evaluation of a novel multiepitope vaccine construct targeting the uPAR. The vaccine candidate incorporates carefully selected immunogenic epitopes derived from the uPAR antigen and a potent TLR4 agonist as an adjuvant. Extensive computational analyses, including antigenicity prediction, allergenicity assessment, physicochemical characterization, and structural modeling, underscore the vaccine’s promising attributes. The molecular docking and dynamics simulations provide valuable insights into the vaccine’s robust interaction with the TLR4 receptor and its structural stability. The computational immune simulation predicted a successful vaccine response characterized by sustained antibody production, robust cellular immunity, and establishment of immunological memory. These findings lay a solid foundation for advancing this multiepitope vaccine toward experimental validation and preclinical studies. The unique design leveraging uPAR’s pivotal role in cancer progression and the immunostimulatory properties of the TLR4 agonist, holds significant potential for developing an effective cancer immunotherapeutic strategy. However, before considering clinical translation, further investigations are warranted to evaluate the vaccine’s immunogenicity, safety, and efficacy in relevant in vitro and in vivo models.

## Ethics Statement

The present study has been confirmed by the Ethics Committee of Hamadan University of Medical Sciences, Hamadan, Iran (Ethical Approval IR.UMSHA.REC.1402.285).

## Consent

The authors have nothing to report.

## Disclosure

All authors read and approved the final version of the manuscript.

## Conflicts of Interest

The authors declare no conflicts of interest.

## Author Contributions

Amir Taherkhani designed the study. Amir Taherkhani, Zahra Hemmat, and Ali Teimoori performed the methods. Amir Taherkhani, Fahimeh Baghaei, Setareh Shojaei, and Ali Teimoori discussed the results. Amir Taherkhani and Ali Teimoori wrote the manuscript. Fahimeh Baghaei and Setareh Shojaei edited the manuscript.

## Funding

This research received no specific grant from any funding agency in the public, commercial, or not‐for‐profit sectors.

## Supporting Information

Additional supporting information can be found online in the Supporting Information section.

## Supporting information


**Supporting Information** Figure S1. Superimposed vaccine structure before and after model refinement. The green chain represents the vaccine model prior to refinement, while the violet chain depicts the structure postrefinement.

## Data Availability

The datasets used and/or analyzed during the current study are available from the corresponding author upon reasonable request.

## References

[1] Kong H. K. and Park J. H. , Characterization and Function of Human Ly-6/uPAR Molecules, BMB Reports. (2012) 45, no. 11, 595–603, 10.5483/BMBRep.2012.45.11.210, 2-s2.0-84871425009. 23186997 PMC4133805

[2] Wang L. , Lin X. , and Sun P. , UPAR, Beyond Regulating Physiological Functions, Has Orchestrated Roles in Cancer (Review), International Journal of Oncology. (2022) 61, no. 6, 10.3892/ijo.2022.5441. 36263620

[3] Wang L. , Yang R. , Zhao L. , Zhang X. , Xu T. , and Cui M. , Basing on uPAR-Binding Fragment to Design Chimeric Antigen Receptors Triggers Antitumor Efficacy Against uPAR Expressing Ovarian Cancer Cells, Biomedicine & Pharmacotherapy. (2019) 117, 10.1016/j.biopha.2019.109173, 2-s2.0-85068029773, 109173. 31387176

[4] Porcelli L. , Guida M. , and De Summa S. , et al.UPAR(+) Extracellular Vesicles: A Robust Biomarker of Resistance to Checkpoint Inhibitor Immunotherapy in Metastatic Melanoma Patients, Journal for Immunotherapy of Cancer. (2021) 9, no. 5, 10.1136/jitc-2021-002372, e002372. 33972390 PMC8112420

[5] Liu M. , Chen S. , Zhang A. , Zheng Q. , and Fu J. , PLAUR as a Potential Biomarker Associated With Immune Infiltration in Bladder Urothelial Carcinoma, Journal of Inflammation Research. (2021) 14, 4629–4641, 10.2147/JIR.S326559. 34552345 PMC8450190

[6] Zhai B. T. , Tian H. , and Sun J. , et al.Urokinase-Type Plasminogen Activator Receptor (uPAR) as a Therapeutic Target in Cancer, Journal of Translational Medicine. (2022) 20, no. 1, 10.1186/s12967-022-03329-3, 135. 35303878 PMC8932206

[7] Wang Z. , Wang K. , Gao X. , Liu Z. , and Xing Z. , Comprehensive Analysis of the Importance of PLAUR in the Progression and Immune Microenvironment of Renal Clear Cell Carcinoma, PLoS ONE. (2022) 17, no. 6, 10.1371/journal.pone.0269595, e0269595. 35675366 PMC9176830

[8] Lourenço A. L. , Chuo S. W. , and Bohn M. F. , et al.High-Throughput Optofluidic Screening of Single B Cells Identifies Novel Cross-Reactive Antibodies as Inhibitors of uPAR With Antibody-Dependent Effector Functions, mAbs. (2023) 15, no. 1, 10.1080/19420862.2023.2184197, 2184197. 36859773 PMC9988344

[9] Hu Y. D. , Wu K. , and Liu Y. J. , et al.LY6/PLAUR Domain Containing 3 (LYPD3) Maintains Melanoma Cell Stemness and Mediates an Immunosuppressive Microenvironment, Biology Direct. (2023) 18, no. 1, 10.1186/s13062-023-00424-3, 72. 37924160 PMC10623712

[10] Chen D. S. and Mellman I. , Oncology Meets Immunology: The Cancer-Immunity Cycle, Immunity. (2013) 39, no. 1, 1–10, 10.1016/j.immuni.2013.07.012, 2-s2.0-84880706152. 23890059

[11] Hinrichs C. S. and Rosenberg S. A. , Exploiting the Curative Potential of Adoptive T-Cell Therapy for Cancer, Immunological Reviews. (2014) 257, no. 1, 56–71, 10.1111/imr.12132, 2-s2.0-84890147150. 24329789 PMC3920180

[12] Palucka A. K. and Coussens L. M. , The Basis of Oncoimmunology, Cell. (2016) 164, no. 6, 1233–1247, 10.1016/j.cell.2016.01.049, 2-s2.0-84960422665. 26967289 PMC4788788

[13] Fridman W. H. , Pagès F. , Sautès-Fridman C. , and Galon J. , The Immune Contexture in Human Tumours: Impact on Clinical Outcome, Nature Reviews Cancer. (2012) 12, no. 4, 298–306, 10.1038/nrc3245, 2-s2.0-84858800620. 22419253

[14] Pardoll D. M. , The Blockade of Immune Checkpoints in Cancer Immunotherapy, Nature Reviews Cancer. (2012) 12, no. 4, 252–264, 10.1038/nrc3239, 2-s2.0-84858766182. 22437870 PMC4856023

[15] Sakuishi K. , Ngiow S. F. , and Sullivan J. M. , et al.TIM3^+^ FOXP3^+^ Regulatory T Cells are Tissue-Specific Promoters of T-Cell Dysfunction in Cancer, Oncoimmunology. (2013) 2, no. 4, 10.4161/onci.23849, 2-s2.0-84885716979, e23849. 23734331 PMC3654601

[16] Schmitz-Winnenthal F. H. , Hohmann N. , and Niethammer A. G. , et al.Anti-Angiogenic Activity of VXM01, an Oral T-Cell Vaccine Against VEGF Receptor 2, in Patients With Advanced Pancreatic Cancer: A Randomized, Placebo-Controlled, Phase 1 Trial, OncoImmunology. (2015) 4, no. 4, 10.1080/2162402X.2014.1001217, 2-s2.0-84954342124, e1001217. 26137397 PMC4485742

[17] Schmitz-Winnenthal F. H. , Hohmann N. , and Schmidt T. , et al.A Phase 1 Trial Extension to Assess Immunologic Efficacy and Safety of Prime-Boost Vaccination With VXM01, an Oral T Cell Vaccine against VEGFR2, in Patients With Advanced Pancreatic Cancer, OncoImmunology. (2018) 7, no. 4, 10.1080/2162402X.2017.1303584, 2-s2.0-85041007678, e1303584. 29632710 PMC5889207

[18] de Paula Peres L. , da Luz FAC. , and dos Anjos Pultz B. , et al.Peptide Vaccines in Breast Cancer: The Immunological Basis for Clinical Response, Biotechnology Advances. (2015) 33, no. 8, 1868–1877, 10.1016/j.biotechadv.2015.10.013, 2-s2.0-84961885009. 26523780

[19] Pol J. G. , Bridle B. W. , and Lichty B. D. , Detection of Tumor Antigen-Specific T-Cell Responses After Oncolytic Vaccination, Oncolytic Viruses. (2020) 2058, 191–211, 10.1007/978-1-4939-9794-7. 31486039

[20] Vermaelen K. , Vaccine Strategies to Improve Anti-Cancer Cellular Immune Responses, Frontiers in Immunology. (2019) 10, 10.3389/fimmu.2019.00008, 2-s2.0-85061151594, 418373. PMC6349827 30723469

[21] Lee S. J. , Shin S. J. , and Lee M. H. , et al.A Potential Protein Adjuvant Derived From *Mycobacterium tuberculosis* Rv0652 Enhances Dendritic Cells-Based Tumor Immunotherapy, PLoS ONE. (2014) 9, no. 8, 10.1371/journal.pone.0104351, 2-s2.0-84905457093, e104351. 25102137 PMC4125215

[22] Peng C. , Tang F. , Wang J. , Cheng P. , Wang L. , and Gong W. , Immunoinformatic-Based Multi-Epitope Vaccine Design for Co-Infection of *Mycobacterium tuberculosis* and SARS-CoV-2, Journal of Personalized Medicine. (2023) 13, no. 1, 10.3390/jpm13010116, 116. 36675777 PMC9863242

[23] Dariushnejad H. , Ghorbanzadeh V. , Akbari S. , and Hashemzadeh P. , Design of a Novel Recombinant Multi-Epitope Vaccine Against Triple-Negative Breast Cancer, Iranian Biomedical Journal. (2022) 26, no. 2, 160–174. 35090304 10.52547/ibj.26.2.160 PMC8987416

[24] Sanami S. , Azadegan-Dehkordi F. , and Rafieian-Kopaei M. , et al.Design of a Multi-Epitope Vaccine Against Cervical Cancer Using Immunoinformatics Approaches, Scientific Reports. (2021) 11, no. 1, 10.1038/s41598-021-91997-4, 12397. 34117331 PMC8196015

[25] Chatterjee N. , Ojha R. , Khatoon N. , and Prajapati V. K. , Scrutinizing *Mycobacterium tuberculosis* Membrane and Secretory Proteins to Formulate Multiepitope Subunit Vaccine Against Pulmonary Tuberculosis by Utilizing Immunoinformatic Approaches, International Journal of Biological Macromolecules. (2018) 118, 180–188, 10.1016/j.ijbiomac.2018.06.080, 2-s2.0-85048839318. 29920369

[26] Deng H. , Yu S. , and Guo Y. , et al.Development of a Multivalent Enterovirus Subunit Vaccine Based on Immunoinformatic Design Principles for the Prevention of HFMD, Vaccine. (2020) 38, no. 20, 3671–3681, 10.1016/j.vaccine.2020.03.023. 32247566

[27] Vita R. , Mahajan S. , and Overton J. A. , et al.The Immune Epitope Database (IEDB): 2018 Update, Nucleic Acids Research. (2019) 47, no. D1, D339–D343, 10.1093/nar/gky1006, 2-s2.0-85059798226. 30357391 PMC6324067

[28] Doytchinova I. A. and Flower D. R. , VaxiJen: A Server for Prediction of Protective Antigens, Tumour Antigens and Subunit Vaccines, BMC Bioinformatics. (2007) 8, no. 1, 1–7, 10.1186/1471-2105-8-4, 2-s2.0-33847031827. 17207271 PMC1780059

[29] Gupta S. , Kapoor P. , Chaudhary K. , Gautam A. , Kumar R. , and Patterson R. L. , In Silico Approach for Predicting Toxicity of Peptides and Proteins, PLoS ONE. (2013) 8, no. 9, 10.1371/journal.pone.0073957, 2-s2.0-84884141339, e73957. 24058508 PMC3772798

[30] Remmert M. , Biegert A. , Hauser A. , and Söding J. , HHblits: Lightning-Fast Iterative Protein Sequence Searching by HMM-HMM Alignment, Nature Methods. (2012) 9, no. 2, 173–175, 10.1038/nmeth.1818, 2-s2.0-84856489442. 22198341

[31] Dimitrov I. , Bangov I. , Flower D. R. , and Doytchinova I. , AllerTOP v.2—A Server for in Silico Prediction of Allergens, Journal of Molecular Modeling. (2014) 20, no. 6, 1–6, 10.1007/s00894-014-2278-5, 2-s2.0-84901583210. 24878803

[32] Magnan C. N. , Randall A. , and Baldi P. , SOLpro: Accurate Sequence-Based Prediction of Protein Solubility, Bioinformatics. (2009) 25, no. 17, 2200–2207, 10.1093/bioinformatics/btp386, 2-s2.0-69949162927. 19549632

[33] Gasteiger E. , Hoogland C. , and Gattiker A. , et al.Protein Identification and Analysis Tools on the ExPASy Server, 2005, Springer.

[34] Zhou X. , Zheng W. , and Li Y. , et al.I-TASSER-MTD: A Deep-Learning-Based Platform for Multi-Domain Protein Structure and Function Prediction, Nature Protocols. (2022) 17, no. 10, 2326–2353, 10.1038/s41596-022-00728-0. 35931779

[35] Seok C. , Baek M. , Steinegger M. , Park H. , Lee G. R. , and Won J. , Accurate Protein Structure Prediction: What Comes Next?, BIODESIGN. (2021) 9, no. 3, 47–50, 10.34184/kssb.2021.9.3.47.

[36] Heo L. , Park H. , and Seok C. , GalaxyRefine: Protein Structure Refinement Driven by Side-Chain Repacking, Nucleic Acids Research. (2013) 41, no. W1, W384–W388, 10.1093/nar/gkt458, 2-s2.0-84883590772. 23737448 PMC3692086

[37] Wiederstein M. and Sippl M. J. , ProSA-Web: Interactive Web Service for the Recognition of Errors in Three-Dimensional Structures of Proteins, Nucleic Acids Research. (2007) 35, no. suppl_2, W407–W410, 10.1093/nar/gkm290, 2-s2.0-34547566446. 17517781 PMC1933241

[38] Laskowski R. , MacArthur M. , and Thornton J. , PROCHECK: Validation of Protein-Structure Coordinates, 2006.

[39] Laskowski R. A. , MacArthur M. W. , Moss D. S. , and Thornton J. M. , PROCHECK: A Program to Check the Stereochemical Quality of Protein Structures, Journal of Applied Crystallography. (1993) 26, no. 2, 283–291, 10.1107/S0021889892009944.

[40] Yan Y. , Zhang D. , Zhou P. , Li B. , and Huang S.-Y. , HDOCK: A Web Server for Protein–Protein and Protein–DNA/RNA Docking Based on a Hybrid Strategy, Nucleic Acids Research. (2017) 45, no. W1, W365–W373, 10.1093/nar/gkx407, 2-s2.0-85023197328. 28521030 PMC5793843

[41] Yan Y. , Tao H. , He J. , and Huang S.-Y. , The HDOCK Server for Integrated Protein–Protein Docking, Nature Protocols. (2020) 15, no. 5, 1829–1852, 10.1038/s41596-020-0312-x. 32269383

[42] Laskowski R. A. , Jabłońska J. , Pravda L. , Vařeková R. S. , and Thornton J. M. , PDBsum: Structural Summaries of PDB Entries, Protein Science. (2018) 27, no. 1, 129–134, 10.1002/pro.3289, 2-s2.0-85039057623. 28875543 PMC5734310

[43] Lopéz-Blanco J. R. , Garzón J. I. , and Chacón P. , IMod: Multipurpose Normal Mode Analysis in Internal Coordinates, Bioinformatics. (2011) 27, no. 20, 2843–2850, 10.1093/bioinformatics/btr497, 2-s2.0-80053988669. 21873636

[44] López-Blanco J. R. , Aliaga J. I. , Quintana-Ortí E. S. , and Chacón P. , iMODS: Internal Coordinates Normal Mode Analysis Server, Nucleic Acids Research. (2014) 42, no. W1, W271–W276, 10.1093/nar/gku339, 2-s2.0-84904811832. 24771341 PMC4086069

[45] Stothard P. , The Sequence Manipulation Suite: JavaScript Programs for Analyzing and Formatting Protein and DNA Sequences, BioTechniques. (2000) 28, no. 6, 1102–1104, 10.2144/00286ir01. 10868275

[46] Grote A. , Hiller K. , and Scheer M. , et al.JCat: A Novel Tool to Adapt Codon Usage of a Target Gene to Its Potential Expression Host, Nucleic Acids Research. (2005) 33, no. suppl_2, W526–W531, 10.1093/nar/gki376, 2-s2.0-23144444226. 15980527 PMC1160137

[47] Ermolaeva M. D. , Khalak H. G. , White O. , Smith H. O. , and Salzberg S. L. , Prediction of Transcription Terminators in Bacterial Genomes, Journal of Molecular Biology. (2000) 301, no. 1, 27–33, 10.1006/jmbi.2000.3836, 2-s2.0-0034604382. 10926490

[48] Bernaschi M. and Castiglione F. , Design and Implementation of an Immune System Simulator, Computers in Biology and Medicine. (2001) 31, no. 5, 303–331, 10.1016/S0010-4825(01)00011-7, 2-s2.0-0034861860. 11535199

[49] K. Lund I. , Illemann M. , Thurison T. , J. Christensen I. , and Hoyer-Hansen G. , uPAR as Anti-Cancer Target: Evaluation of Biomarker Potential, Histological Localization, and Antibody-Based Therapy, Current Drug Targets. (2011) 12, no. 12, 1744–1760, 10.2174/138945011797635902, 2-s2.0-80054086465. 21707477

[50] Mazar A. P. , The Urokinase Plasminogen Activator Receptor (uPAR) as a Target for the Diagnosis and Therapy of Cancer, Anti-Cancer Drugs. (2001) 12, no. 5, 387–400, 10.1097/00001813-200106000-00001, 2-s2.0-0034933664. 11395568

[51] Montuori N. , Pesapane A. , and Rossi F. W. , et al.Urokinase Type Plasminogen Activator Receptor (uPAR) as a New Therapeutic Target in Cancer, Translational Medicine @ UniSa. (2016) 15, 15–21. 27896223 PMC5120746

[52] Oh H. A. , Lee G. , and Kang H. J. , et al.Overexpression of c-met Protein in Gastric Cancer and Role of uPAR as a Therapeutic Target, Cancer Research and Treatment. (2003) 35, no. 1, 9–15, 10.4143/crt.2003.35.1.9. 26680909

[53] Soria-Guerra R. E. , Nieto-Gomez R. , Govea-Alonso D. O. , and Rosales-Mendoza S. , An Overview of Bioinformatics Tools for Epitope Prediction: Implications on Vaccine Development, Journal of Biomedical Informatics. (2015) 53, 405–414, 10.1016/j.jbi.2014.11.003, 2-s2.0-84924341672. 25464113

[54] Childers N. K. , Miller K. L. , and Tong G. , et al.Adjuvant Activity of Monophosphoryl Lipid A for Nasal and Oral Immunization With Soluble or Liposome-Associated Antigen, Infection and Immunity. (2000) 68, no. 10, 5509–5516, 10.1128/IAI.68.10.5509-5516.2000, 2-s2.0-0033805905. 10992447 PMC101499

[55] Cluff C. W. , Jeannin J. F. , Monophosphoryl Lipid A (MPL) as an Adjuvant for Anti-Cancer Vaccines: Clinical Results, Lipid A in Cancer Therapy, 2010, 667, Springer, New York, NY, 111–123. 10.1007/978-1-4419-1603-7_10 20665204

[56] Didierlaurent A. M. , Morel S. , and Lockman L. , et al.AS04, an Aluminum Salt-and TLR4 Agonist-Based Adjuvant System, Induces a Transient Localized Innate Immune Response Leading to Enhanced Adaptive Immunity, The Journal of Immunology. (2009) 183, no. 10, 6186–6197, 10.4049/jimmunol.0901474, 2-s2.0-77249176352. 19864596

[57] Steinhagen F. , Kinjo T. , Bode C. , and Klinman D. M. , TLR-Based Immune Adjuvants, Vaccine. (2011) 29, no. 17, 3341–3355, 10.1016/j.vaccine.2010.08.002, 2-s2.0-79954582124. 20713100 PMC3000864

[58] Shirota H. , Tross D. , and Klinman D. , CpG Oligonucleotides as Cancer Vaccine Adjuvants, Vaccines. (2015) 3, no. 2, 390–407, 10.3390/vaccines3020390, 2-s2.0-85016863945. 26343193 PMC4494345

[59] Chen X. , Zaro J. L. , and Shen W.-C. , Fusion Protein Linkers: Property, Design and Functionality, Advanced Drug Delivery Reviews. (2013) 65, no. 10, 1357–1369, 10.1016/j.addr.2012.09.039, 2-s2.0-84881018686. 23026637 PMC3726540

[60] Reddy Chichili V. P. , Kumar V. , and Sivaraman J. , Linkers in the Structural Biology of Protein–Protein Interactions, Protein Science. (2013) 22, no. 2, 153–167, 10.1002/pro.2206, 2-s2.0-84874033858. 23225024 PMC3588912

[61] Khalid K. , Irum S. , Ullah S. R. , and Andleeb S. , In-Silico Vaccine Design Based on a Novel Vaccine Candidate Against Infections Caused by *Acinetobacter baumannii* , International Journal of Peptide Research and Therapeutics. (2022) 28, no. 1, 1–17, 10.1007/s10989-021-10316-7. PMC8636788 34873398

[62] Hasan M. and Mia M. , Exploratory Algorithm of a Multi-Epitope-Based Subunit Vaccine Candidate Against *Cryptosporidium hominis*: Reverse Vaccinology-Based Immunoinformatic Approach, International Journal of Peptide Research and Therapeutics. (2022) 28, no. 5, 10.1007/s10989-022-10438-6, 134. 35911179 PMC9315849

[63] Makrides S. C. , Strategies for Achieving High-Level Expression of Genes in *Escherichia coli* , Microbiological Reviews. (1996) 60, no. 3, 512–538, 10.1128/mr.60.3.512-538.1996. 8840785 PMC239455

