# Supplementary material for: Rational Design and In Silico Evaluation of a Multiepitope Vaccine Targeting the uPAR for Cancer Immunotherapy
Source: J Immunol Res. 2025 Oct 16;2025:9126083. doi: 10.1155/jimr/9126083 (PMC12531433; doi:10.1155/jimr/9126083)
Supplement: Supplementary file 1 — Supporting Information Figure S1. Superimposed vaccine structure before and after model refinement. The green chain represents the vaccine model prior to refinement, while the violet chain depicts the structure postrefinement. [file JIMR-2025-9126083-s001.docx]

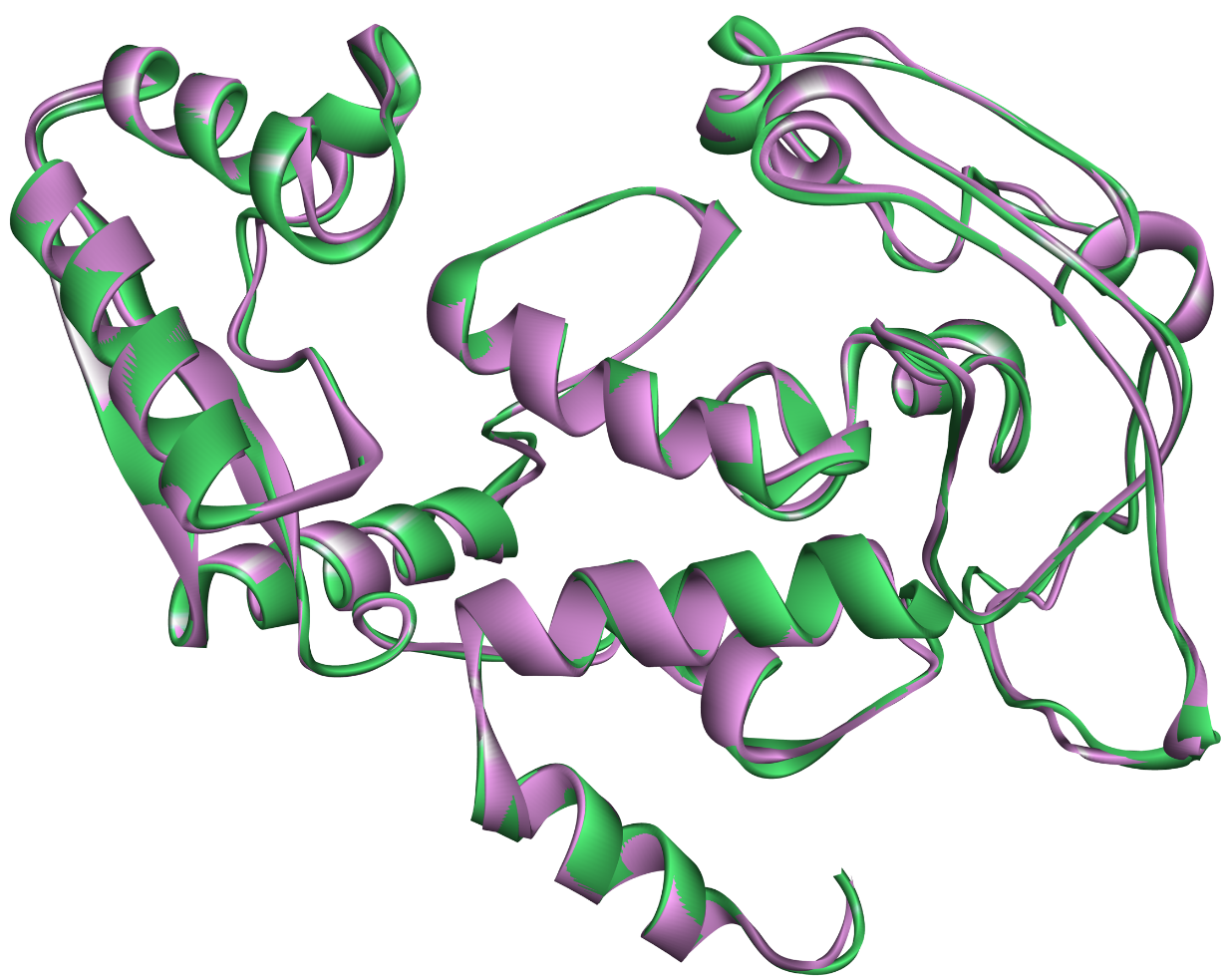


**Supplementary Figure 1.** Superimposed vaccine structure before and after model refinement. The green chain represents the vaccine model prior to refinement, while the violet chain depicts the structure post-refinement.
